# Measles prevention in adolescents: lessons learnt from implementing a high school catch-up vaccination programme in New South Wales, Australia, 2014–2015

**DOI:** 10.5365/WPSAR.2016.7.1.009

**Published:** 2016-09-01

**Authors:** Sonya Nicholl, Holly Seale, Vicky Sheppeard, Sue Campbell-Lloyd

**Affiliations:** a Immunisation Unit, Health Protection New South Wales, Australia.; b School of Public Health and Community Medicine, UNSW Australia, New South Wales, Australia.; c Communicable Diseases Branch, Health Protection New South Wales, Australia.

## Abstract

**Introduction:**

In response to a significant increase of measles cases and a high percentage of unvaccinated adolescents in New South Wales, Australia, a measles high school catch-up vaccination programme was implemented between August and December 2014. This study aimed to explore the factors affecting school-based supplementary immunization activities (SIAs) and to inform future SIA and routine school-based vaccination programme implementation and service provision.

**Methods:**

Focus group analysis was conducted among public health unit (PHU) staff responsible for implementing the SIA catch-up programme. Key areas discussed were pre-programme planning, implementation, resources, consent materials, media activity and future directions for school vaccination programme delivery. Sessions were audio recorded, transcribed verbatim and reviewed. Thematic analysis was conducted to identify the major themes.

**Results:**

Two independent focus groups with 32 participants were conducted in January 2015. Barriers to the SIA implementation included lead time, consent processes, interagency collaboration, access to the targeted cohort and the impact of introducing a SIA to an already demanding curriculum and school programme immunization schedule. A positive PHU school coordinator rapport and experience of PHU staff facilitated the implementation. Consideration of different approaches for pre-clinic vaccination status checks, student involvement in the vaccination decision, online consent, workforce sharing between health districts and effective programme planning time were identified for improving future SIA implementation.

**Conclusion:**

Although many barriers to school programme implementation have been identified in this study, with adequate resourcing and lead time, SIAs implemented via a routine school vaccination programme are an appropriate model to target adolescents.

## Introduction

In March 2014, the World Health Organization announced that measles elimination had been achieved in Australia. (*1*) While this is a significant accomplishment for public health in Australia, consistent high measles vaccination coverage of over 95% for a single dose and over 90% for two doses for each new birth cohort is required to achieve herd immunity and maintain measles elimination. (*2*)

Measles elimination does not mean the absence of the disease, rather it signifies the absence of ongoing local measles transmission. Due to measles’ highly infectious nature, the non-immune status of many young adult travellers was seen as a risk to maintaining elimination. Many of the 40 cases notified in New South Wales (NSW) in early 2014 were associated with overseas travel or contact with those who had recently returned from countries such as the Philippines, Indonesia and other parts of Asia where large measles outbreaks were occurring. (*3*) Teenagers and young adults are a high-risk cohort because they may have missed vaccination and/or the second dose was not recommended in the National Immunization Schedule during their childhood. (*4*) They are also of an age when travel to countries with endemic measles is common.

In 2014, it was determined that almost 40% of NSW teenagers were recorded as not fully vaccinated against measles on the Australian Childhood Immunization Register (ACIR). (*5*) The rate of full vaccination was lowest among senior high school students, while junior high school students had acceptable levels in most districts. Ongoing measles transmission in NSW was also noted to be associated with young adult travellers. (*3*) Consequently, a supplementary immunization activity (SIA), the NSW Measles High-school Catch-up Vaccination Program, was delivered between August and December 2014. The SIA was implemented by NSW public health unit (PHU) staff, located in 15 local health districts (LHDs), with an essential role in delivering the routine annual school vaccination programme to students in their first year of high school.

In May 2014, PHUs were asked to select schools in their district where high numbers of unvaccinated students were expected to attend. The aim was to prioritize offering vaccination to senior high school students before they completed their schooling at the end of 2014. (*6*, *7*) A media campaign was conducted which included a PHU hotline and dedicated web site. A parent information kit was developed which included a parental recall section regarding their child’s measles, mumps and rubella (MMR) vaccination history. Of the 90 800 students enrolled in the 145 targeted schools, parental consent was provided for over 19 000 (21%) students and over 11 000 (12%) were vaccinated.

While school-based vaccination programmes have been implemented by NSW Health since 2003, there has never been a study conducted to explore the attitudes and perceptions of NSW PHU staff towards school vaccination services. This study aimed to explore the delivery of immunization through time-limited SIAs and to identify factors affecting their success.

## Methods

A qualitative study involving focus groups was undertaken in January 2015. An invitation to participate was sent to PHU staff who were involved in school vaccination programme administration and implementation. The focus groups were scheduled to coincide with their annual immunization professional development day.

An interview guide was developed by the investigators to identify key areas of interest for the study. Questions covered key areas which included pre-programme planning, implementation, resources, consent materials, media activity and future directions for school vaccination programme delivery. Written informed consent was obtained, and participants were randomly assigned to a focus group using *Random Number Generator* (GraphPad Software Inc., La Jolla, CA, USA) software. The focus groups were facilitated by a senior policy analyst in the NSW Health Immunization Unit. At the end of the sessions, the facilitator summarized and reported participants’ views to the group to ensure they were accurately recorded.

The focus group sessions were audio recorded, transcribed verbatim and reviewed as a whole along with field notes. To find repeated patterns of meaning across all data sets, repeated reading, coding and thematic analysis was undertaken by one coder. A proportion of the data (30%) was coded by an independent coder and the findings were compared with the initial results for data validation. Text was organized within the identified themes of the developed framework using NVIVO Version 10 (QSR International Pty Ltd, Victoria, Australia).The results were presented according to the major themes that were identified.

Ethics approval was obtained from the University of New South Wales Human Research Ethics Medical Panel (HREA PANEL Reference: 2014–7-66) for this study.

## Results

Two independent focus group discussions were conducted in January 2015 with 32 staff from 15 PHUs. There were 30 female and two male participants whose occupations included immunization coordinators, school programme coordinators, nurses and team leaders and administrative support staff. The majority of the participants were highly experienced with over five years’ experience in school programme planning and implementation (24/32, 75%) (**Table 1**). The duration of the two focus group discussions was 60 minutes and 50 minutes, respectively.

**Table 1:** Characteristics of the subjects that participated in the school-based SIA focus group sessions, NSW, Australia, 2015

Characteristics	n	%
**Sex**		
Male	2	6.3%
Female	30	93.8%
**Level of experience for school vaccination programme**		
High	24	75.0%
Medium	6	18.8%
Low	2	6.3%
**Position**		
District Immunization Coordinator	12	37.5%
School Programme Coordinator	7	21.96%
School Programme Team Leader	4	12.5%
School Programme Registered Nurse	4	12.5%
Administrative support	5	15.6%
**Total**	**32**	**100%**

### Identifying the target schools

Participants spoke about difficulties in accurately identifying their target schools. Several participants thought that the ACIR data provided to identify schools were inaccurate as many of the children had relocated. This made it difficult to select the high priority schools. Others commented that offering the programme in all LHDs concerned them as they believed that the programme should only have focused on specific metropolitan areas where there had been recent measles outbreaks. One participant described it as “*just drawing straws basically to do it*.” Only one participant thought that the data were useful to select the schools and confirm the knowledge of MMR coverage in the area.

### Identifying and vaccinating eligible students

When asked about performing the pre-clinic ACIR checks, one participant advised that to not do so would be a *“waste of the health dollar”* and that as a *“registered nurse costs 44 Australian dollars per hour, putting them in a school for quite a while”* to vaccinate students, many of whom do not need to be vaccinated, was deemed *“wasteful.”* For one PHU, it was reported that of the 400 consent forms distributed to a school, 100 students were consented; however, post-ACIR checks revealed that only 21 required vaccination. Another participant reported that *“80% of students did not need to be vaccinated”* after ACIR checking. The checks however were considered to be a *“huge imposition”* as it was reported that many school programme staff did not have ACIR data access and different student details on the consent form made it difficult to identify them on the register.

Many reported that access to Year 12 students was very restricted by the schools due to impending Higher School Certificate trials and exams: *“We could not get to Years 11 and 12. We did Years 9 and 10 because the majority of schools … wouldn’t let us in for 11 and 12” and “… they asked us to rethink our cohort ... and because we had to ... we chose a younger group.”* On the other hand, it was agreed by many that if some students received a third dose of MMR vaccine due to inaccurate parental recall, this was more acceptable than delaying the clinic and potentially losing the opportunity to vaccinate this high-risk cohort.

### New approaches to informing parents

The majority of participants reported the hotline as a useful initiative for new vaccination programmes. However, many felt that the message was long at just over three minutes and was thought to interfere with other PHU calls. Participants were very positive towards the pre-programme coverage in newspapers and on social media sites; however, some commented that reading newspapers was less common and recommended more social media activity. They all agreed that the measles campaign web site was *“absolutely essential”* to refer callers to when introducing new vaccination programmes.

### Strategies to engage parents and schools

Nearly all participants agreed that the parent information kit content was generally *“easy and straightforward”*; however, one felt that an information kit specific for Aboriginal people would have been beneficial. In some areas, the nurses delivered the kits to the schools that they identified as a positive networking opportunity to build a rapport with school staff and address any concerns. One PHU sent the kits directly to parents in an attempt to improve consent rates.

Some participants believed that many students completed the consent form and asked their parent to *“just sign it”* and that it was the students who made the vaccination decision, which participants identified as a major factor for the kits reaching the parents to provide consent for their child to be vaccinated.

### Validity of parent recall

Participants reported that parents were “confused” by the parental recall section on the consent form. Nearly all agreed that the ‘two dose’ box on the form was superfluous as many parents ticked it and signed the form to have their child vaccinated. Parental confusion regarding previously documented doses of MMR was thought to be due to vaccination records documenting Priorix, which was not identified as an MMR vaccine. Many agreed that, because of this, several children may have received a third dose of MMR vaccine. To counteract this, one PHU distributed a letter to parents that documented the MMR vaccine brands.

### School coordination

The pre-clinic ACIR checks resulted in a much lower number of students being vaccinated than were consented and was reported as *“wasteful”* and *“unappreciated”* by some school staff as they had planned their clinic according to consented student numbers. Additionally, some school year coordinators considered the SIA an *“inconvenience”* due to interruptions to the curriculum, particularly at short notice. However, a previous measles outbreak in a school correlated with a positive acceptance of the SIA. Conversely, feedback was reported from several unsatisfied parents of students attending non-targeted schools as they were required to attend their primary care physician for vaccination which was viewed as an *“inconvenience.”*

### Workforce

Introducing the SIA at short notice was reported to compound staffing arrangements for the routine school programme. A shared casual pool of nurse immunizers was suggested for future SIAs. Concerns were raised about prioritizing the SIA against the ever increasing competing demands on PHU immunization coordinators. *“We have lots of things that are taking our focus hugely now, and it’s getting bigger and bigger ... the resourcing needs to be looked at really as to what are the priorities…”*

It was argued however that if only one non-immunized student was vaccinated then it would be *“extremely worthwhile”* as *“just one infectious person with measles can contaminate many more.”*

Informing school staff about the programme was reported as a challenge in areas with multiple immunization teams. Furthermore, improved communication from NSW Health to primary care physicians (about school vaccination services) was recommended. One participant said, *“I spoke with a [physician] last night because we were doing a measles clinic ... and he thought [the vaccine] wasn’t available free for them.”*

### Inter-agency collaboration

In this SIA, PHUs were required to liaise with school coordinators with whom they had no previous contact or professional relationship (as the routine programme involves only Year 7 students). Those who reported a positive PHU-school coordinator relationship identified a positive impact on student access and clinic planning. Those with a less positive rapport described it as a *“struggle,”* particularly with the limited planning period.

## Discussion

Although in one study, routine school-based vaccination programmes were found to be successful in facilitating high vaccination coverage of a cohort that do not routinely access medical services; (*8*) adding MMR vaccine to the routine school programme does not guarantee high uptake in unvaccinated adolescents when compared to an SIA targeting the same group. Future SIAs should continue to be targeted at the at-risk cohort.

SIAs have made a significant contribution towards the successful elimination of measles in the European Region. (*9*) In the United Kingdom of Great Britain and Northern Ireland, a school catch-up programme offered MMR vaccine to targeted school-leavers in 12 high schools in conjunction with a school leavers’ vaccinations programme. It was concluded that this model was logistically convenient and may reduce the extent of future outbreaks. (*10*) Some previous measles SIAs implemented in NSW have specifically targeted at-risk ethnic groups, while others were outbreak control initiatives conducted by physicians or PHU staff. (*11*-*13*) While there has never been a state-wide school-based SIA implemented and examined in NSW, this study has highlighted factors affecting school-based SIAs and has shown that future SIAs implemented via a routine school vaccination programme could be an appropriate model to target adolescents.

For identifying and vaccinating eligible students, we found that there was confusion among PHU staff regarding the purpose and mechanisms of the programme. For example, some did not understand that the most recent recorded residential addresses were used to identify postcode areas with low vaccination coverage. In the future, more time should be spent ensuring that PHU staff fully understand the data and imperatives underpinning an SIA. The location of adolescent SIAs also needs to be carefully considered. If the school setting is selected, clinic scheduling needs to be considered, particularly to maximize access to students in the final year.

Despite its short lead time and duration, this SIA successfully vaccinated over 11 000 (12%) students enrolled in the targeted schools. It is unlikely such a high uptake would have been achieved using an alternative model. (*11*, *14*) A physician-delivered community programme in the United Kingdom in 2013 vaccinated 10.77% (95% CI: 6.97–14.57) of the targeted unvaccinated population; however, heterogeneity in coverage was identified. (*15*) The United Kingdom study concluded that efforts should have been focused on populations with low coverage rather than implementing national campaigns. This is in line with our study’s finding that many of the consented students did not need to be vaccinated. While some PHU staff expressed concern about delivering a state-wide SIA, the associated supports, such as mass media, programme web site and hotline, were seen as facilitating uptake. However, effective public communication support is only achievable if SIAs are coordinated across the state and not feasible if PHUs undertake ad hoc catch-up programmes.

For the concerns reported by some school coordinators about PHUs vaccinating fewer students than had consented, it could be addressed in future programmes by including routine communication to the school coordinator before the clinic day. On the other hand, the process of requiring parents to return consent forms needs to be reviewed to maximize vaccine uptake. (*16*) A study revealed that a more reliable method for distribution of consent forms, along with pre-campaign educational programmes, was needed along with prior notice of the programme and suitable venue. (*17*) Online parental consent could be pursued; however, how equitable access can be maintained and how parental consent can be verified should be considered before system implementation.

A well established school vaccination programme can overcome many barriers such as cost, access and time for parents. (*16*) Effective planning is essential, (*18*) and a school’s commitment to the vaccination clinics has also been found to effect the pre-vaccination logistics. (*19*) The SIA in this study was conducted over a short time period as it needed to be scheduled in the last two school terms of the academic year, which influenced the planning timeframe. Despite the short lead time, this SIA was deemed to be successful due to the experience of PHU staff in planning and delivering school-based programmes and positive school coordinator attitudes and rapport with the PHU.

Implementing a time-limited, school-based SIA at short notice is a challenge that requires an innovative approach to engage parents and students. A study from the United States of America found that parents of adolescents have competing priorities and poor participation rates in a school vaccination programme were related to busy parents; some parents had limited knowledge and language skills to consent for their child to be vaccinated. (*20*) Another study found that adolescents have an increasing role in decision-making regarding vaccinations and that parents respect their child’s right to refuse to be vaccinated. (*21*) A theme of ‘joint decision-making’, between students and their parents has been identified as an influencing factor in decision-making for school-based human papillomavirus vaccination of adolescents. (*22*, *23*) Educating students could encourage them to advocate for parental consent and reduce anxiety. (*16*) Although teachers have no obligations for school vaccination programme education, (*8*) a student resource, such as an advice card with appropriate language and graphics explaining the importance of the vaccine, is recommended.

In this SIA, parental consent depended on their recall of their child’s previous measles vaccinations. However, when conducted, ACIR pre-clinic checks found parental recall to be inaccurate. A similar study also found underreporting of vaccinations through parental recall. (*24*) Provider validation of parent-reported vaccinations is required to ensure accurate surveillance of vaccination coverage of adolescents. One school vaccination programme (*25*) presumed that the risks of undervaccination exceeded the risks of over-vaccination. If parents were unsure about their children’s vaccination status but consented for vaccination, students were vaccinated. It is known that approximately 5% of recipients fail to seroconvert to their first dose of MMR vaccine. (*26*) Given poor parental recall and incomplete MMR vaccine seroconversion, the parental recall section on the consent form should be removed in future school-based SIAs to facilitate vaccination of all consented students without pre-programme ACIR checks.

The PHU staff members were targeted for the focus group discussions as they are essential school programme facilitators who communicate with school staff, immunization teams, parents and students. They were highly representative of the NSW school vaccination programme workforce. Focus groups were considered more logistically feasible than individual interviews with similar research outcomes due to participants’ location, availability and time constraints. In this study, 16 participants in each focus group exceeded the recommended six to 10 participants for a standard focus group interview; (*27*) however, we believe that exceeding this recommendation did not negatively impact on the discussions. While participants were randomly assigned to each group, it was difficult to ensure an even mix of personalities and experience; however, each group was manageable, and participation of each participant was equally high and engaging in general. Meanwhile, the facilitator who had a key responsibility for coordinating this SIA, had regular contact with the cohort regarding non-school-based vaccination programme matters. This could have influenced the responses of participants in the interviews and should be noted.

## Conclusion

Future SIAs should be carefully considered regarding their lead time, location, targeted year group, available resources and workforce. The benefits of implementing the SIA through an already established programme by experienced staff outweigh the disadvantages. With adequate resourcing and lead time, SIAs implemented via a routine school vaccination programme are an appropriate model to target adolescents.
